# Biological and clinical impact of central nervous system hemangioblastomas in Chinese patients with von Hippel-Lindau disease: implications for treatment

**DOI:** 10.1186/s13053-020-00153-y

**Published:** 2020-10-22

**Authors:** Zhen Liu, Liang Li, Zhiqiang Yi, Hongzhou Duan, Runchun Lu, Chunwei Li, Lei Li, Kan Gong

**Affiliations:** 1 Department of Neurosurgery, Peking University First Hospital, No. 8 Xishiku Street, Xicheng District, Beijing, 100034 China; 2 Department of Urology, Peking University First Hospital, Beijing, 100034 China

**Keywords:** VHL genetic syndrome, Genetic mutation, Hemangioblastoma surgical treatment, Genetic anticipation

## Abstract

**Objective:**

Central nervous system (CNS) hemangioblastomas (HGBs) are the most frequent cause of mortality in patients with von Hippel-Lindau (VHL) genetic syndrome. However, there is a lack of large studies on the clinical features and optimal management of HGBs in Chinese patients.

**Methods:**

VHL-related HGB cases treated surgically at our hospital from 2012 to 2019 were evaluated. Patients and family members meeting the clinical diagnostic criteria underwent genetic testing. Clinical, genetic and relevant imaging data were analyzed.

**Results:**

Eighty-five VHL patients from 34 pedigrees in 16 Chinese provinces who underwent 121 operations for CNS HGBs were enrolled. Multiple operations were associated with a younger age at first operation (OR = 0.926, 95% CI = 0.871–0.985, *P* = 0.014, threshold: 27.5, sensitivity: 72.2%, specificity: 71.2%) and a longer postoperative period (OR = 1.096, 95% CI = 1.015–1.184, *P* = 0.019, threshold: 10.5, sensitivity: 66.7%, specificity: 76.3%). The age at first operation was younger in children than in their parents (23 pairs, *P* < 0.001). The age at first operation was younger in siblings born later than in those born earlier **(**10 pairs, *P* = 0.01). Most untreated tumors (98.2%) remained relatively stable during follow-up (range, 0.5–7; median, 2). However, new tumors continued to emerge (0.14 tumor/year).

**Conclusion:**

VHL-associated CNS HGB is a long-term chronic disease with repeated attacks, likely with genetic anticipation in Chinese pedigrees. When the age at first operation is under 27.5 years, or the postoperative period is longer than 10.5 years, the risk of multiple operations is increased. While most unresected HGBs remain stable after surgery, new tumors may still slowly emerge; hence, scheduled follow-ups are necessary.

Von Hippel-Lindau (VHL) genetic syndrome is an autosomal dominant genetic disease caused by pathogenic variants in the VHL tumor suppressor gene on chromosome 3 (3p25–26). It consists of three exons and two introns within a 10 kb region [1, 2]. This disease carries a prevalence of 1 in 36,000 births and is highly penetrant (> 90% penetrance by 65 years of age) [3, 4]. The *VHL* gene encodes a tumor suppressor protein (pVHL) that regulates hypoxia-inducible factor (HIF) protein. The main reason for VHL genetic syndrome is pVHL inactivation caused by genetic mutations, resulting in dysfunction in regulating the proteolytic degradation of HIF [5]. Uncontrolled HIF expression increases the expression of a wide range of target genes involved in angiogenesis, proliferation and metabolism, including vascular endothelial growth factor (VEGF) and C-X-C motif chemokine receptor 4 (CXCR4). VEGF overexpression is a leading factor in tumor angiogenesis, especially for hemangioblastomas (HGBs) [6, 7]. The main manifestation of VHL syndrome in the central nervous system (CNS) is HGB. Although HGB is a benign tumor, its cystic characteristics and associated peritumoral edema often lead to neurological morbidity and mortality [8]. Additionally, its natural history and management have not been defined. In recent studies, researchers have considered the tumor burden of VHL-associated HGBs to be associated with partial germline mutations, male sex and a younger age [9–11]. Most of these studies were performed in European and North American countries. There is a lack of large studies on the clinical features, pathogenic variants and optimal management of HGBs in Chinese patients. Therefore, we retrospectively analyzed patients with VHL-associated CNS HGBs admitted between 2012 and 2019. The patients and family members who met the clinical diagnostic criteria underwent genetic testing. Clinical, genetic and relevant imaging data were analyzed.

## Methods

### Patients

This study was approved by the ethics committee of our hospital. All patients involved in this study provided informed consent. From June 2012 to May 2019, 37 patients with VHL genetic syndrome confirmed by genetic diagnosis underwent surgery for CNS HGBs at our hospital, and all cases were verified by postoperative pathology. These patients who met the clinical diagnostic criteria and their family members underwent genetic testing. The diagnosis was established when the patient was found to carry a germline *VHL* mutation or fulfilled the previously described clinical criteria. Because of the late onset in some patients, their biological parents were deceased and not available for genetic testing. At least one patient was diagnosed via *VHL* mutation testing in each family; Clinical data including sex, family history, mutation type, onset age of each organ, the cause of death and relevant imaging data were obtained by reviewing medical records or interviewing family members. Fiften patients were excluded from the study due to unclear data. A total of 85 VHL patients from 34 pedigrees in 16 Chinese provinces who underwent 121 operations for CNS HGBs were enrolled.

### Germline genotype analysis

Genomic DNA was extracted from peripheral blood using the QIAamp DNA Blood Mini Kit (QIAGEN, Germany). Three coding exons and their flanking intronic regions were amplified by polymerase chain reaction (PCR) using primers as described previously [12]. Direct sequencing was used to detect missense and splicing mutations. Large exon deletions were detected using a multiplex ligation-dependent probe amplification kit (MLPA, P016-C2, MRC-Holland, Amsterdam) and confirmed by real-time quantitative PCR using the primers described by Ebenazer et al. [13]. All patients were divided into the following five groups: missense group, partial deletion group, large deletion group, frameshift group and splicing site mutation group.

### Clinical assessment and imaging analysis

All surgical patients underwent contrast-enhanced MRI (T1-weighted imaging (T1WI), T2-weighted imaging, diffusion-weighted imaging and contrast-enhanced T1WI; slice thickness, 1 mm) examinations to determine the lesion location. The volume of the tumor and associated cysts was calculated using a modified ellipsoid formula (volume = largest anteroposterior diameter (centimeter)*largest mediolateral diameter (centimeter)*largest craniocaudal diameter (centimeter)*0.5) [14]. Abdominal computed tomography and fundoscopy were performed in all patients to identify related lesions in other target organs. The Karnofsky Performance Scale (KPS) score was used to evaluate neurological function, and postoperative follow-up examinations were performed every 6 months. Patients underwent follow-up contrast-enhanced MRI examinations to detect the presence of unresected tumor or recurrence.

### Surgical technique

Surgery was performed on patients who had tumors with obvious mass effects on MRI. Because HGBs are usually located in the posterior and medial portions of the cerebellum, brainstem and spinal cord, we often used the posterior median approach for surgical resection. For tumors located anteriorly, laterally or at other sites, we selected the appropriate surgical approach based on optimum visualization and direct access to the tumor. The process of tumor resection was typically performed as follows: First, we separated the tumor from the surrounding tissue. Then, we gradually severed the feeding arteries and drainage veins. Finally, we completely removed the tumor. For tumors with high-pressure peritumoral cysts, we released the cystic fluid to relieve the pressure and then removed the tumor. After complete tumor removal, the cyst was reexamined for residual tumor. For large cysts, we used endoscopy to explore the cysts to avoid missing tumors. Preoperative embolization was not performed in any patient.

### Statistical analysis

Patients and tumor characteristics were analyzed using descriptive statistics. The difference in the number of operations in each variant group was compared using the Mann-Whitney U test. The chi-squared test was used to compare the sex distribution between two groups. The significance of associations with the outcome of multiple operations was evaluated using univariate logistic analysis. Then, multivariate analysis was used to analyze those significant variables as independent predictors for multiple operations. Receiver operating characteristic (ROC) curve analysis was used to evaluate the predictive value. The optimal cutoff value was calculated by Youden’s index. Differences between generations and peers were analyzed using the Wilcoxon test.

## Results

### Patient characteristics

A total of 85 VHL patients from 34 pedigrees in 16 Chinese provinces who underwent 121 operations for CNS HGBs were enrolled. The mean age at first operation in these patients was 33.9 ± 13.6 years (range, 13–74; median, 30). The male-to-female ratio was 1.02 (males, 43; females, 42). Genetic analysis showed that there were 13 families in the partial deletion group, 3 families in the large deletion group, one family in the frameshift group and 15 families in the missense group (Table 1). Variants within exons appeared in 32 pedigrees. Two types of splicing stie variants appeared in 2 families. Because of the late onset in some patients, their biological parents were deceased and not available for genetic testing. However, 8 (8/33, 24%) of the remaining patients exhibited de novo mutations confirmed by paternity testing. According to the American College of Medical Genetics and Genomics standards and guidelines, we found that there were 68 pathogenic variants and 17 likely pathogenic variants among these patients.

**Table 1:** Patient characteristics in different variants type

	Families	HGB patients(total)	HGB patients (alive)	Male	First operation age in HGB	The average times of operation in HGB
Partial deletion	13	35	33	23	32.8 ± 14.3	1.3 ± 0.8
Large deletion	3	12	10	5	34.8 ± 12.8	1.8 ± 1.4
Frameshift	1	1	1	1	39	1
Missense	15	31	27	20	33 ± 12.3	1.3 ± 0.8
Splicing site mutation	2	6	6	2	40.2 ± 19.1	1.7 ± 1.2
total	34	85	77	43	33.7 ± 13.6	1.4 ± 0.93
*P*	–	–	–	0.593	0.716	0.628

**Fig. 1:**
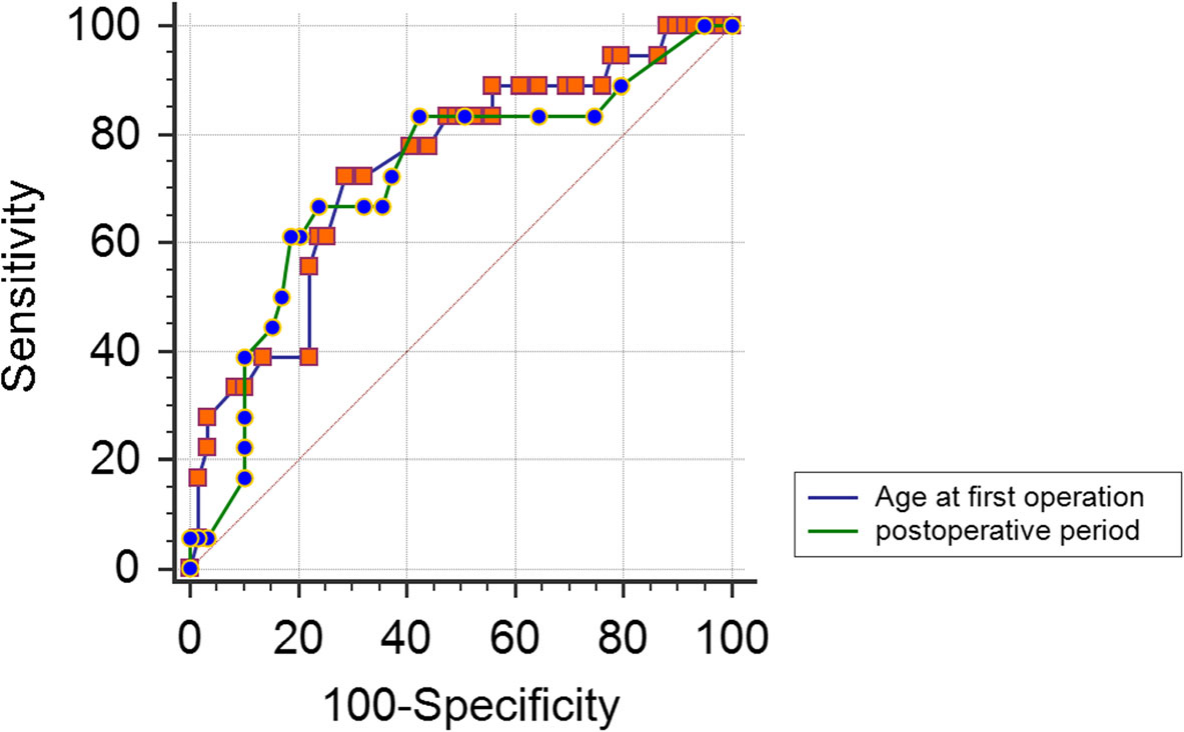
ROC curve for multiple operations according to the age at first operation and the period after the first operation

Eight patients died from aspiration and infection after the first operation. The remaining 77 patients (males, 38; females, 39; age, 43 ± 14.7; range, 18–76; median, 43) recovered well after the first operation and survived until December 2019. Forty-five of the 77 patients in the deletion group (including partial deletions, large deletions and a frameshift group) underwent 66 operations for CNS HGB, and 33 patients in the missense group underwent 48 operations. Our statistical results showed no significant difference in sex, age at first operation or number of operations among five kinds of variants (Table 1). The chi-square test results showed no significant difference in the number of operations between the deletion group and missense group (*P* = 0.824). In the multivariate analysis, only two covariates remained in the final model: the age at first operation (OR = 0.926, 95% CI = 0.871–0.985, *P* = 0.014 < 0.05) and the period after the first operation (OR = 1.096, 95% CI = 1.015–1.184, *P* = 0.019 < 0.05). The probability of reoperation was calculated using the following formula: log(p/1-p) = 0.092 × 1 + 0.077 × 2(χ2 = 15.462 *P* < 0.001) (Table 2). Model performance was used to select thresholds to discriminate patients by age at first operation and period after the first operation. As shown in Fig. 1, the area under the ROC curve (AUC) of the selected model for age at first operation was 0.737 (95% CI = 0.624–0.831, standard error: 0.069). The optimal threshold was 27.5 (sensitivity: 72.2%, specificity: 71.2%, Youden’s index: 0.434). The AUC of the selected model for the period after the first operation was 0.718 (95% CI = 0.575–0.860, standard error: 0.073), and the optimal threshold was 10.5 (sensitivity: 66.7%, specificity: 76.3%, Youden’s index: 0.43). The difference between the two AUCs was not significant (*P* = 0.84). In addition, genealogical analysis showed that the age at first operation was younger in children than in their parents (23 pairs, *P* < 0.001). This phenomenon might be related to genetic anticipation. The age at first operation was younger in siblings born later than those born earlier (10 pairs, *P* = 0.01 < 0.05).

**Table 2:** Analysis of factors associated with number of operations

Variables	*P* value	OR,95% CI
Sex	0.254	–
Mutation	0.261	–
Age at first operation	0.14	0.926,0.871–0.985
Period after the first operation	0.19	1.096,1.015–1.184

### Tumor characteristics

**Fig. 2:**
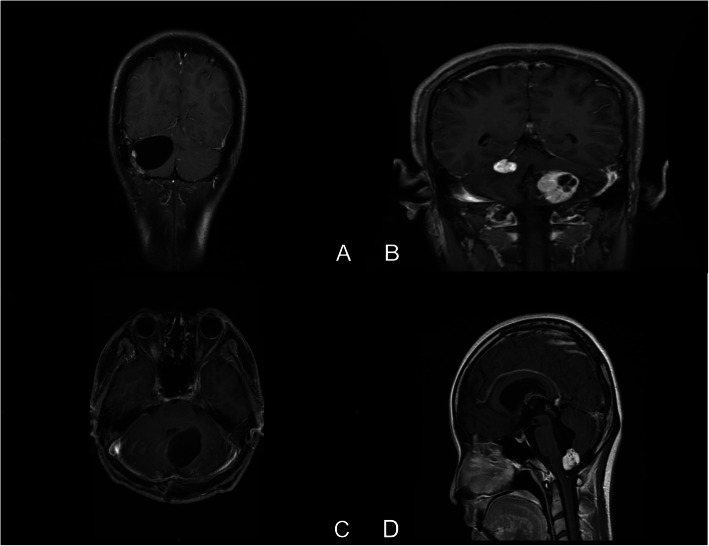
Postcontrast MRI of cerebellar HGB and peritumoral cyst (**a** and **c**), solid cerebellar tumor and intratumoral cyst (**b**), and HGB in the medulla oblongata (**d**)

Because some of the patients did not undergo surgery at our hospital, to accurately assess the tumor burden, distribution and development, we analyzed the clinical and relevant imaging data from only patients who underwent surgery at our hospital. The mean operative age of the 37 patients treated at our hospital was 36.3 ± 13.6 years (range, 20–67 years; median, 34 years), the male-to-female ratio was 0.95 (males, 18; females, 19), and the mean follow-up duration was 2.24 ± 1.71 years (range, 0.5–7 years; median, 2 years). Preoperative MRI showed that 37 patients harbored 182 CNS HGBs, with an average of 4.92 ± 2.92 tumors/patient (range, 1–13; median, 4). The tumor sites were as follows: 66 tumors (36%), right cerebellum; 52 tumors (29%), left cerebellum; 8 tumors (4%), cerebellar vermis; 19 tumors (10%), medulla oblongata; 23 tumors (13%), cervical spinal cord; and 14 tumors, other sites (Fig. 2). The major symptoms of HGBs in the cerebellum are headache (40.5%) and gait ataxia (45.9%). Symptomatic HGBs in the brainstem could lead to hydrocephalus (45.9%) causing intracranial hypertension. Symptomatic tumors in the spine and cauda equina could lead to pain or urinary/bowel abnormalities. Out of the enrolled patients, only one patient with a symptomatic tumor of the thoracic spine underwent surgical intervention.

Thirty-seven patients underwent 40 operations at our hospital; 71 CNS HGBs were removed, with an average of 1.78 ± 1.02 tumors/patient, all of which were confirmed by postoperative pathology (range, 1–6; median, 2). Of these tumors, 51 were located in the cerebellum, 6 were located in the cervical spinal cord, 1 was located in the thoracic cord, and 13 were located in the medulla oblongata. Thirty-one cystic tumors were resected, including tumors with peritumoral and intratumoral cysts. Most of the cysts were located in the dorsal part near the midline. Thirty cystic lesions were located in the cerebellum, and 1 was located in the cervical spinal cord. The mean volume of cystic lesions resected from the cerebellum was 12.6 ± 10.1 cm^3^. All resected tumors in the medulla oblongata were solid. Due to the obvious mass effects in the medulla oblongata, these 7 HGBs were resected to avoid aggravating the hydrocephalus. The mean volume of tumors in the medulla oblongata was 9.2 ± 7.3 cm^3^. Most of the other tumors were removed in the same operative field. Although the space occupying effects were not obvious, they were near those of the larger tumors. To prevent the postoperative volume from increasing, the other tumors were concurrently removed. Tumors not in the same operative area and without obvious occupying effects were not treated at the same time.

Thirty-six patients recovered well postoperatively and were discharged approximately 1 week later. One patient died from aspiration after surgery. Because of the high recurrence and low growth rate, our follow-up interval was 6 months. The mean follow-up duration in these 36 patients was 2.24 ± 1.71 years (range, 0.5–7; median, 2). The KPS score at 6 months postoperatively was 94.7 ± 7.7 (range, 80–100; median, 100). Postoperative reexamination showed that 109 (98.2%) of the remaining 111 tumors were in a stable state. Tumor enlargement was found in only 2 cases (1.8%): one was located in the thoracic spinal cord, with obvious spinal cord compression, and reoperation was performed; the other was in the cerebellum without obvious symptoms, and this patient continued to be followed by observation. In addition, 10 new cerebellar HGBs appeared in 5 patients, for a recurrence rate of 0.14 tumors/year. Among the 37 patients, 81.1% had pancreatic cysts, 48.6% had renal cysts, and 48.6% had renal cancer. Pheochromocytomas were found in 13.5% of patients. Retinal HGBs occurred in 27% of patients. Thirty-seven patients underwent a total of 115 operations for renal cancer, pheochromocytomas, retinal HGBs and CNS HGBs, with an average of 3.1 ± 1.59 operations/patient (range, 1–7; median, 3).

## Discussion

### Biological and clinical implications

Due to the pathogenic variants in *VHL*, CNS HGBs might manifest as a long-term chronic disease with repeated attacks occurring in VHL patients. In our study, 85 of the 99 VHL patients (85.8%) from 34 pedigrees in 16 Chinese provinces underwent surgery for CNS HGBs. These 85 patients underwent a total of 121 operations for CNS HGBs (with a mean (SD) number of operations per patient of 1.47 (0.97)). These findings are consistent with the results of other research. In reports from the United States and Italy, over 80% of VHL patients develop CNS HGBs during their lifetime, and more than 90% of patients with VHL-related HGBs develop multiple CNS HGBs [15, 16].

According to the public Human Gene Mutation Database (HGMD: http://www.hgmd.org), 627 variants have been identified. Missenses and partial deletions are the most common pathogenic variants in *VHL*. In our investigation, of 99 patients from 34 different families, 56% showed deletions, and 44% showed missenses; 24% (8/33) of these mutations were identified as de novo mutations, similar to the results reported by the NIH. We found no significant difference between the deletion groups (17 families, 44 HGB patients) and the missense groups (15 families, 33 HGB patients) in the number of operations (*P* = 0.876), the number of CNS HGBs (*P* = 0.208) or tumor size (cerebellum, *P* = 0.643; medulla oblongata, *P* = 0.380). The number of operations for renal cell carcinoma (RCC) (*P* = 0.827) or retinal angioma (RA) (*P* = 0.285) were also not significantly different between the deletion and missense groups. However, some studies have considered that partial deletions are associated with a higher risk of developing a large number of CNS HGBs [9, 17]. Perhaps this is because the protein produced by missense germline alterations can maintain intrinsic function in the context of neoplasias and metabolic syndromes, including *VHL* [18, 19]. However, other studies have shown that deletions might be related to a lower risk of RCC and RA development in VHL patients [20]. Furthermore, Franke et al. observed that germline deletions were associated with a higher risk of RA development [17]. However, according to the present findings, there can be no unified conclusions regarding the exact mechanisms underlying some VHL manifestations. For this reason, it is difficult to determine the relationship between mutations and tumorigenesis. Further research is required to determine the mechanisms by which *VHL* pathogenic variants influence phenotypes.

In addition, the progression of CNS HGBs was related to a younger age; our results reveal that age is a risk factor for tumor progression. A younger age at first operation for CNS HGBs results in a higher risk of reoperation. According to our statistical analysis, an age at first operation younger than 27.5 years predicted reoperation with a sensitivity and specificity of 72.2 and 71.2%, respectively. Additionally, a longer period after surgery was associated with a higher chance of reoperation; a period longer than 10.5 years predicted reoperation for CNS HGBs, with a sensitivity and specificity of 66.7 and 76.3%, respectively. Some studies have found that younger patients are much more likely to develop more tumors per year than older patients [9]. Hence, the early resection and close follow-up of symptomatic CNS HGBs are very important for patients with a younger age of onset. Simultaneously, the results from the family study show that the age at first operation for CNS HGBs was younger in children than in their parents (23 pairs, *P* = 0.0 < 0.001). The regularity with which this occurs might be associated with genetic anticipation. The reasons for this phenomenon are considered to be related to telomere shortening in the offspring [21]. Our results also show that the age at first operation was younger in siblings born later than in those born earlier. The cause of this phenomenon remains unclear. Some scholars have considered the cause to be related to hormone exposure during pregnancy [22].

### Tumor burden and treatment

Most VHL patients (92%) often have multiple CNS HGBs resected when undergoing surgery on the nervous system, with an average of 4.92 ± 2.92 CNS HGBs/patient. More than half (67%) of the tumors were located in the cerebellum. The chief mechanism responsible for the symptomatology is the cystic changes in solid tumors. Nearly 84% of patients underwent surgery due to the mass effects caused by cystic tumor changes. Some studies have considered that cystic tumors grow faster than the mean associated tumor growth rate and that symptoms caused by cysts in highly eloquent areas may be related to early cyst progression [23]. The mechanism of peritumoral cyst formation mainly involves high intratumoral pressure and increased vascular permeability [24]. Currently, the treatment of cystic tumors is primarily surgical resection. Recent studies showed that radiotherapy for peritumoral cysts achieves limited effects and could result in peritumoral edema or even cyst progression [25]. Therefore, early surgical resection seems to be an effective therapy for symptomatic CNS HGB cysts. Furthermore, our results show that solid tumors located in the medulla oblongata are more likely to lead to hydrocephalus in the early stage. However, the very complex areas and abundant vascular networks make tumor resection difficult. Approximately 10% of the tumors were located in the medulla. Additionally, 32% of medullary HGBs were resected due to hydrocephalus. The mean volume of tumors resected from the medulla oblongata was 9.2 ± 7.3 cm^3^. Because of the abundant blood supply in the tumor, some surgeons apply preoperative embolization to reduce the risk of bleeding. However, none of the 37 patients treated at our hospital underwent preoperative embolization. Only 2 patients (5%) received intraoperative blood transfusions, and postoperative hemorrhage was not observed in any patient. Except for one patient who died from aspiration, all 36 patients recovered well, with no complications. The 6-month postoperative follow-up yielded a mean KPS score of 94.7 ± 7.7 (range, 80–100; median, 100). Recent studies have also reported that preoperative embolization did not decrease the blood loss volume or incidence of postoperative complications; additionally, preoperative embolization fails to improve the gross total resection rate, and the embolization procedure carries a significant risk for complications [26–28]. Postoperative reexaminations revealed that most tumors (98.2%) were relatively stable, with only 2 tumors increasing in volume. Ten new-onset cerebellar HGBs were found in 13.5% of the patients, showing an average new tumor rate of 0.14 tumors/year. Considering the growth pattern of CNS HGBs, it is not necessary for patients to undergo frequent follow-up examinations; the follow-up interval for CNS HGBs should be 1 year or 6 months.

## Conclusion

VHL-related CNS HGB is a long-term chronic disease with repeated attacks, likely with genetic anticipation in Chinese pedigrees with VHL-related HGBs. The age at first operation is younger in siblings born later than in those born earlier. Symptoms of HGBs are mainly related to the mass effects caused by cystic changes, and the most common site is the cerebellum. When the age at first operation is under 27.5 years or the operation interval is longer than 10.5 years, the risk of multiple operations is increased. Most unresected HGBs are stable after surgery; however, new tumors may continue to emerge. Hence, scheduled follow-up examinations are necessary.

## Data Availability

Not applicable.
